# Gut-lung axis and dysbiosis in COVID-19

**DOI:** 10.3906/biy-2005-102

**Published:** 2020-06-21

**Authors:** Busra AKTAS, Belma ASLIM

**Affiliations:** 1 Department of Molecular Biology and Genetics, Faculty of Arts and Sciences, Burdur Mehmet Akif Ersoy University, Burdur Turkey; 2 Department of Biology, Faculty of Science, Gazi University, Ankara Turkey

**Keywords:** COVID-19, gut-lung axis, dysbiosis, gut permeability

## Abstract

COVID-19, a novel infectious disease, caused by SARS-CoV-2, affected millions of people around the world with a high mortality rate. Although SARS-CoV-2 mainly causes lung infection, gastrointestinal symptoms described in COVID-19 patients and detection of the viral RNA in feces of infected patients drove attentions to a possible fecal-oral transmission route of SARS-CoV-2. However, not only the viral RNA but also the infectious viral particles are required for the viral infection and no proof has been demonstrated the transmission of the infectious virus particles via the fecal-oral route yet. Growing evidence indicates the crosstalk between gut microbiota and lung, that maintains host homeostasis and disease development with the association of immune system. This gut-lung interaction may influence the COVID-19 severity in patients with extrapulmonary conditions. Severity of COVID-19 has mostly associated with old ages and underlying medical conditions. Since the diversity in the gut microbiota decreases during aging, dysbiosis could be the reason for older adults being at high risk for severe illness from COVID-19. We believe that gut microbiota contributes to the course of COVID-19 due to its bidirectional relationship with immune system and lung. Dysbiosis in gut microbiota results in gut permeability leading to secondary infection and multiple organ failure. Conversely, disruption of the gut barrier integrity due to dysbiosis may lead to translocation of SARS-CoV-2 from the lung into the intestinal lumen via circulatory and lymphatic system. This review points out the role of dysbiosis of the gut microbiota involving in sepsis, on the severity of SARS-CoV-2 infection. Additionally, this review aims to clarify the ambiguity in fecal-oral transmission of SARS- CoV-2.

## 1. Introduction

A novel coronavirus called severe acute respiratory syndrome coronavirus 2 (SARS- CoV-2) was detected in China and affected millions of people around the world with a very high mortality rate (Y.C. Wu et al., 2020; Zhu et al., 2020), much more than the severe acute respiratory syndrome coronavirus (SARS-CoV) and the Middle East respiratory syndrome coronavirus (MERS-CoV) combined (Mahase, 2020). The disease, with the symptoms of fever and dry cough, caused by the current coronavirus was named as COVID-19 by WHO and declared as pandemic due to the ongoing global health crisis which SARS- CoV-2 led to (World Health Organization, 2020; Zhu et al., 2020). Several coronavirus species have been identified that cause human diseases including SARS-CoV, MERS-CoV, and the current coronavirus, SARS- CoV-2. Human coronaviruses mostly have a long single-stranded RNA with positive polarity (Hilgenfeld and Peiris, 2013). SARS- CoV-2 virions have a double layer lipid envelope structure on the outer surface with various proteins such as spike (S) protein, envelope (E) protein, and membrane (M) protein embedded in (X. Li et al., 2020). Phylogenetic analysis of complete genome sequences of SARS-CoV-2 revealed that the new virus shares 89.1% nucleotide sequence identity with SARS-like coronaviruses detected in bats. SARS-CoV-2 is closely related to SARS-CoV but more distant to MERS (Elfiky, 2020; A. Wu et al., 2020; F. Wu et al., 2020). SARS-CoV-2 RNA dependent RNA polymerase, which has been used as target for antiviral inhibitors, shares 90.1% sequence identity to SARS-CoV but 56.7% that of to MERS-CoV (Elfiky, 2020). While MERS-CoV uses dipeptidyl peptidase 4 (DPP4), SARS-CoV and SARS-CoV-2 use the angiotensin-converting enzyme 2 (ACE2) to enter human cells. It has been shown that receptor affinity of SARS-CoV-2 to ACE2 is higher than the strain of SARS-CoV in 2003 (Wan et al., 2020). Although the SARS-CoV-2 has spread more quickly compared to SARS-CoV and MERS-CoV due to possible increased globalization, they do not seem very different regarding their course of disease and there are several similarities in their clinical signs (Peeri et al., 2020). The manner of development of this current infectious disease has not been uncovered yet and there is not a comprehensive explanation of the pathogenesis for COVID-19; however, the mechanism of SARS-CoV and MERS-CoV can give us quite valuable information on the pathogenesis of SARS-CoV-2 infection. Although the COVID-19 mortality has been seen in patients with varying age, older adults are still at the higher risk groups (Xu et al., 2020). Studies to date have mostly associate the disease severity with elders and patients who have underlying medical conditions. However, the contribution of leaky gut to the severity of COVID-19 due to dysbiosis in the gut microbiota has not been revealed clearly. This review points out the role of dysbiosis of the gut microbiota involving in sepsis, on the severity of SARS- CoV-2 infection. Additionally, this review aims to clarify the ambiguity in fecal-oral transmission of SARS- CoV-2.

## 2. Gastrointestinal symptoms of COVID-19 and hypotheses propounded

The main symptoms at onset of illness in COVID-19 patients from Wuhan were defined as fever, cough, and fatigue by the retrospective reports and the symptoms such as headache, diarrhea, vomiting, and abdominal pain were described as less common symptoms due to low incidence (N. Chen et al., 2020; Huang et al., 2020; Zhu et al., 2020). However, as more information collected from different regions and large scale studies, the incidence of the patients with gastrointestinal (GI) symptoms has been increased (Holshue et al., 2020; Pan et al., 2020; D. Wang et al., 2020). Due to the common symptoms, indicating respiratory tract disease, of patients infected with SARS-CoV-2, the main organ effected by the COVID-19 seems to be lung. However, during the course of the disease, development of dysfunction in organs such a liver and intestine or multiple organ failure has been reported (N. Chen et al., 2020; D’Amico et al., 2020; Feng et al., 2020; Pan et al., 2020; D. Wang et al., 2020). Later on, fecal samples collected from COVID-19 patients were found to be positive of SARS-CoV-2 nucleic acid (Y. Wu et al., 2020). Despite clearance of viral RNA in the respiratory tract, the fecal samples of COVID-19 patients remained positive for about 11 days. The centers performing research on gut microbiota and fecal microbiota transplantation (FMT), therefore, suggested to screen FMT donors for SARS-CoV-2 and drew attention to possible fecal-oral transmission of the virus (Y. Chen et al., 2020; Holshue et al., 2020; T. Zhang et al., 2020; Y. Zhang et al., 2020). This may suggest us a widespread immunopathology or presence of extrapulmonary SARS-CoV-2 infection. Previous studies reported that patients with SARS (2002) representing common symptoms similar to COVID-19 had diarrhea with 16–73% (World Health Organization, 2003). This ratio ranged from 2% to 20% in the patients with COVID-19 (N. Chen et al., 2020; Pan et al., 2020; Xiao et al., 2020; J. jin Zhang et al., 2020). Although the ratio vary among reports, which is probably due to the diagnosing criteria used for diarrhea in different hospitals, we should not underestimate gastrointestinal signs along with the respiratory symptoms (Liang et al., 2020). It was suggested in some studies that SARS-CoV-2 associated diarrhea could appear even before the respiratory symptoms (Lin et al., 2020; Song et al., 2020). Pan et al. investigating the clinical characteristics of COVID-19 with 204 patients documented digestive symptoms including diarrhea, vomiting, and abdominal pain in 50.5% of the patients (Pan et al., 2020). Some of the patients showed only the digestive symptoms. Moreover, they reported that gastrointestinal symptoms were declared more often among the patients, as the severity increased with a higher level of liver enzyme and lower monocyte count in patients with digestive symptoms.

During SARS-CoV-2 infection process, spike protein of the virus recognizes ACE2 receptor to invade host cell via type II transmembrane serine protease (TMPRSS2) (H. Zhang et al., 2020). These 2 proteins need to be coexpressed in the same cell for the virus to be able to enter the host cell. Zhang et al. examined different cell types for ACE2 and TMPRSS2 expression pattern with single-cell transcriptome analysis (H. Zhang et al., 2020). They found that these 2 proteins are mainly coexpressed in lung tissue as well as epithelial and gland cells in esophagus and enterocytes in the ileum and colon. In addition to the data detecting viral RNA in feces previously, these results suggest that SARS-CoV-2 infection does not remain with the respiratory tract only and the gastrointestinal system contribute to the course of the disease as well. However, with the limited data until today, it is hard to propose a fecal-oral transmission route to explain the enteric symptoms in COVID-19 patients and claim that SARS-CoV-2 pass through stomach and reach intestine to infect the intestinal cells as enteric viruses accomplish. Xia et al. examined stool, urine, and serum of 73 patients infected with SARS-CoV-2 throughout their hospitalization for the viral RNA (Xiao et al., 2020). Additionally, they investigated the viral nucleocapsid and ACE2 in gastrointestinal tissues from 1 patient by histologic staining. They detected ACE2, viral RNA and nucleocapsid in epithelium of esophagus, stomach, duedonum, and rectum and suggested fecal-oral transmission as an additional route for the virus to spread the body. These results are notable to understand the mechanism of action SARS-CoV-2 involved in; however, it would be too strong referring to a SARS-CoV-2 infection in gastrointestinal cells due to limitation on sample size, only 1 patient. Moreover, as mentioned above, for the virus to be able to enter the cell, both ACE2 and TMPRSS2 need to be expressed in the cell; it is, therefore, not quite possible to conclude viral entrance to the cell by evaluating only the ACE2 expression. Studies often infer SARS-CoV-2 infection in gastrointestinal system through fecal-oral transmission mostly based on tissue distribution of ACE2 and fecal shedding of SARS-CoV-2 RNA (Y. Chen et al., 2020; D’Amico et al., 2020; Ding and Liang, 2020). However, viral RNA detection in some tissues and feces of COVID-19 patients may support the viral RNA load in the area but not the infectious particles. It could be possible but it requires further investigation and more data. Overall, it is not clear whether enteric symptoms or intestinal injury is the result of direct viral infection, inflammation associated or drug induced damage.

## 3. Gut dysbiosis 

The human gastrointestinal tract hosts over 1014 cells made up of 500 to 1000 bacterial species, which are referred to as the gut microbiota (Gill et al., 2006; Ostaff et al., 2013). There is a well-balanced bidirectional interaction between the microbiota and the immune system. While the microbiota plays a fundamental role in development and maturation of the immune system, the immune system shapes the microbiota composition and functions. Disruption of this balance can lead to human diseases (Gill et al., 2006; Proctor, 2011; Ostaff et al., 2013). Studies on the composition of intestinal microbiota in animals and humans of varying different health conditions suggest that gut microbiota is a major determinant in health and disease, impacting immunity, digestion, and pathogenesis. It also serves as an organ participating in physiological and homeostatic functions (Schrezenmeir and de Vrese, 2001; Gill et al., 2006; Proctor, 2011; Ostaff et al., 2013). Intestinal microbiota, therefore, has found to be disturbed as the other functions in the elderly and diet that strengthen the gut epithelial integrity against pathogens and possible infections increasing immunosenescence has been suggested to elder adults (Salazar et al., 2017). Diversity in the gut microbiota decreases during aging and microbiome composition altered to an imbalance state, so called dysbiosis, leads to immune dysfunction and generalized inflammation (Aleman and Valenzano, 2019). Dysbiosis considered as an indicator of unhealthy microbiome has been linked to various chronic conditions such as asthma, arthritis, obesity, and type 2 diabetes (Tai et al., 2015; Aleman and Valenzano, 2019; Hufnagl et al., 2020). People ≥ 65 years old have found to be at higher risk of death from COVID-19 than those of <65 years old (Ioannidis et al., 2020). The incidence of diarrhea in COVID-19 patients and the high mortality rate in elderly patients, taken together, point to a possible involvement of gut-lung axis in COVID-19 with association of dysbiosis. These may imply that directed alteration of gut microbiota could be utilized to prevent or treat unhealthy conditions in humans. Gut microbiota are also relevant to metabolic syndromes such as obesity and insulin resistance (Bäckhed et al., 2004; Vrieze et al., 2012). Alterations in microbial composition may contribute to the development of metabolic diseases such as liver cirrhosis, nonalcoholic fatty liver disease, and diabetes (Vrieze et al., 2012; Minemura and Shimizu, 2015). Changes in gut permeability impact the liver disease severity via intestinal and systemic inflammation and lead to bacterial infections (Bajaj, 2019). Metabolic diseases are associated with a decrease in gut epithelial integrity and an increased bacterial translocation from the lumen to the mucosa inducing systemic inflammation (Burcelin et al., 2012). Moreover, gut microbiota and intestinal barrier have found to be disrupted in pulmonary diseases (Rutten et al., 2014; Hanada et al., 2018). COVID-19 patients with digestive symptoms tend to experience liver injury more than those of without digestive symptoms (Pan et al., 2020). Dysbiosis, the perturbation of the healthy/normal gut microbiota, has been involved in a wide range of disorders or diseases as mentioned above. This explains why an antibiotic induced imbalance in the gut microbiota can cause diarrhea. Therefore, staying in balance is vital for health and this interaction between the gut microbiota and the immune system has a crucial importance in nonhealthy conditions that could increase COVID-19 severity with extrapulmonary damages.

## 4. Gut-lung axis in COVID-19

Coronaviruses are thought to cause damages in lung and lead pneumonia with imbalanced and hyper-immune responses (X. Li et al., 2020). COVID-19 has been found to cause adverse outcomes related to immune response. Increased proinflammatory cytokines and lymphocytopenia are associated with severe SARS-CoV-2 infection (Huang et al., 2020; Zheng et al., 2020). Enhanced cytokine and chemokine production tend to contribute to “cytokine storm” which lead to severe acute respiratory syndrome in lung and multiple organ failure (Huang et al., 2020; Kalantar-Zadeh et al., 2020). A retrospective study on mortality of COVID-19 with 150 patients from Wuhan, China suggest that COVID-19 mortality might be due to virally induced hyper-inflammation, so called cytokine storm (Ruan et al., 2020). They reported that there were significant differences between death group and discharge group in their blood analysis such as white blood cell counts, lymphocyte counts, and IL-6 level, and moreover, 16% of death had secondary infections. Previously, SARS was characterized by excessive immune response with elevated Th2 cytokines in patients with fatal infections (C. K. Li et al., 2008). SARS coronaviruses have found to infect immune cells in addition to lung epithelium as the primary injury site and hyper-reaction has an important role in immune damage and pathogenesis of the virus (Gu et al., 2005). Immune responses induced by other viral infections such as influenza drive changes in gut microbiota resulting in dysbiosis and increase gut permeability, which may cause secondary infection, bacterial pneumonia (J. Wang et al., 2014). The high levels of circulating proinflammatory cytokines by viral infections are capable of altering gut microbiota and disturbing intestinal integrity. A malfunction in small intestine leads to an altered gut microbiota and inflammation due to the well-balanced bidirectional interaction between the gut microbiota and the immune system. Increased inflammation in intestine leads to a leaky gut allowing bacterial antigens and toxins to translocate to the systemic circulation, further worsening the septic state of the patients with COVID-19. An inflammatory reaction, then, could be initiated leading to multiple organ failure (H. Wang et al. Ma, 2008). In a study with critically ill patients treated in an intensive care unit, multiple organ failure has found to be associated with increased intestinal permeability (Doig et al., 1998). Decrease in the gut barrier integrity involves in translocation of bacteria and inflammatory product to distant organs via intestinal lymphatics and leads to sepsis and multiple organ failure syndrome (Deitch, 2012). Another reason for altered gut microbiota could be use of massive amount of antibiotics. A large number of patients in China received antibiotic therapy with 58–71% during COVID-19 treatment (N. Chen et al., 2020; Guan et al., 2020). Antibiotics result in dysbiosis and increase susceptibility to new infections and inflammatory disorders. Additionally, they can cause antibiotic associated diarrhea (AAD). AAD is a very common side effect of antibiotic usage and results from an imbalance in gut microbiota due to antibiotic use. Moreover, AAD in hospitalized patients can cause *Clostridium difficile* associated diarrhea (CDAD), and conversely, CDAD may cause AAD (Hickson, 2011). Microbial translocation with the decrease in intestinal barrier integrity is followed by a secondary infection. The bacterial translocation from the gut to the lungs has been reported in sepsis and acute respiratory distress syndrome due to a possible barrier dysfunction (Dickson et al., 2017). It is known that the gut and the respiratory tract have been linked to modulate immune responses and dysbiosis in gut microbiota contributes to disease pathogenesis in respiratory tract (Fanos et al., 2020). It has been shown that chronic lung diseases, such as chronic inflammatory lung disease and asthma, are usually developed with GI tract diseases including inflammatory bowel syndrome (IBD) and patients of those have the structure and function of their intestinal mucosa and permeability altered (Roussos et al., 2003; Rutten et al., 2014). Furthermore, it has been reported that around 50% of IBD patients of adults have an impaired lung function with no respiratory disease history (Keely et al., 2012). Vice versa, infectious viruses could translocate from infected lung to distant organs via systemic circulation. Gu et al. studied the pathogenesis of SARS on patients infected with SARS-CoV as well as on autopsies of dead patients using in situ hybridization and electron microscopy (Gu et al., 2005). Monocytes and lymphocytes in blood vessels of lungs as well as circulating white blood cells, lymph nodes, and lymphoid tissues were found to be positive for the viral sequences. Moreover, they found pathological modifications in the digestive tract tissues and refer that immune cells infected by the viruses could circulate and invade the enteric cells resulting in gastrointestinal damage. These results suggest that the coronavirus could translocate to systemic circulation after damage in lung tissue and migrate to intestinal cells through circulatory and lymphatic system. In this manner, the virus can avoid the harsh environment of the gastrointestinal tract with gastric and intestinal fluid that can disrupt the lipid envelop of the virus and inhibit its infectivity. Taken together all this, we can think about other way of reaching to the intestine for SARS-CoV-2, rather than fecal-oral transmission implying ingesting the viral infectious agent (Whittier, 2017). For a successful infection through fecal-oral transmission, virus must deal with biological barriers such as acid in the stomach and bile salts in the intestine after ingestion. Darnell et al. found SARS-CoV to be inactivated under acidic conditions, which is pH<3, suggesting that the virus can be deactivated by gastric secretion after ingestion unless consuming with a large meal that can buffer the acidic environment (Darnell et al., 2004). Pratelli et al. working with canine coronavirus reported complete inactivation of the virus under pH 2.26 and pH 4.38 at 37 °C (Pratelli, 2008). There is almost no information about the survival of SARS-CoV-2 under acidic conditions. In one of the preprints, Sun et al. studied the survival of the SARS-CoV-2 under various environmental conditions such as wet, dry, and acidic (Sun et al., 2020)Preprint. Although the virus survives under wet or dry conditions for up to 3 days, it can survive at pH 2.2 for up to 1 h only at high concentrations. Other than the gastric drawback, the virus needs to avoid bile salts to be able to infect the intestinal cells after ingestion. Bile salts are one of the various mechanisms used in host defense with its detergent action on lipid portion of infectious agent (Bertók, 2004). Viruses are divided into subgroups based on presence or absence of a lipid envelope surrounding the nucleocapsid. Bile salts are very effective on viruses that have lipoprotein envelop while those without envelop are resistant to bile acid. SARS-CoV-2 contains outer lipid-containing membrane and is one of the enveloped viruses (X. Li et al., 2020). Although viral RNA has been detected in fecal samples from COVID-19 patients (Y. Wu et al., 2020; Xiao et al., 2020), no evidence has been demonstrated the transmission of the infectious virus via the fecal-oral route yet. Recently, Zang et al. published a remarkable study on SARS-CoV-2 infection of enterocytes and impact of gastric and intestinal fluid on SARS-CoV-2 (Zang et al., 2020). The results showed that although the viruses are capable of entering the intestinal cells, they are not able to survive in the digestive tract due to gastric fluid with low pH and intestinal environment with bile and digestive enzymes. Moreover, no infectious virus was recovered from the fecal samples of COVID-19 patients. This supports that it is quite early to mention about fecal-oral route and enteric infection of SARS-CoV-2 with limited data we have to date and requires more experimental data about it. However, we should not underestimate the contribution of the gut microbiota to the severity of COVID-19 due to its bidirectional relationship with immune system and being the possible reason of gut permeability resulting in secondary infection and multiple organ failure. 

## 5. Conclusions

As a result, various hypothesis could be introduced to explain the gastrointestinal symptoms in COVID-19 patients; however, very little is known due to the sudden spike in COVID-19 cases and the further research studies are required to make the final conclusion. Detection of the viral RNA in feces but not the viral particles causing infection does not quite support the idea of fecal-oral transmission. We think that gut-lung axis involves in COVID-19 severity with the association of dysbiosis (Figure). Disruption of the gut barrier integrity due to dysbiosis may lead to translocation of SARS-CoV-2 from the lung into the intestinal lumen via circulatory and lymphatic system. This may explain the detection of the virus in feces rather than fecal-oral transmission route. In addition, since the diversity in the gut microbiota decreases during aging, dysbiosis could be the reason of older adults being at high risk for severe illness from COVID-19. Considering the studies on coronaviruses to date and the relationship between the gut microbiota and the immune system, we assume that altering the gut microbiota back to its normal state before the disturbance might be an alternative strategy to alleviate the symptoms of COVID-19 and to shorten the recovery period. In this review, role of the lung and the gut microbiota on COVID-19 was investigated and the importance of further research to fully understand the gut-lung axis in severity of COVID-19 was emphasized.

**Figure 1:**
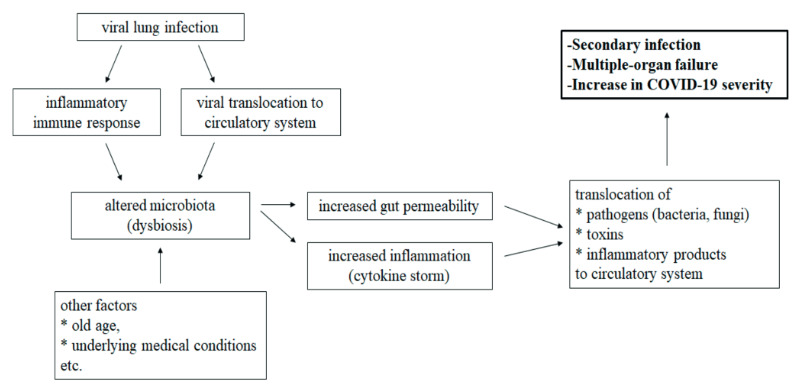
Schematic illustration of gut-lung axis involving in COVID-19 severity with the association of dysbiosis.
